# Data ownership judgments in childhood

**DOI:** 10.1186/s41235-026-00711-3

**Published:** 2026-02-22

**Authors:** Shaylene E. Nancekivell, Abby Vanstone, Kazuki Nishikiori

**Affiliations:** Department of Psychology, University of Manitoba, 190 Dysart Rd, Winnipeg, MB R3T 2N2 Canada

**Keywords:** Data ownership, Digital thinking, Children, Rights, Social cognition

## Abstract

As adults, we experience discomfort when we hear about how *our* data has not been safeguarded by apps. Despite this being a common experience, the psychological processes that underlie people’s reasoning about data ownership and rights are poorly understood. Using a developmental approach, the current investigation examines the psychology of data ownership. We test the specific proposal that people have a coherent theory of ownership that leads them to view data as owned and under owners’ control at the same developmental time point. In both studies (N = 218), children ages 5–12 were told about a user who shares personal and general information with an app. Children then had to decide who it belongs to (Study 1) and who is in charge of it (Study 2). During middle childhood, children view users as owners of personal information and therefore entitled to control it suggesting coherence in their representations of non-physical property types. Findings also suggest that by 8-years-old children have the cognition in place to think of data as property and therefore understand their data rights online.

A quick Google search will offer numerous examples of data breaches involving the unconsented sharing of personal data online (e.g., Facebook data breach). As adults, we experience discomfort when we hear about how *our data* has not been safeguarded. This discomfort stems from the intuition that such violations are violations of our data ownership rights.

In online contexts, attributions of data ownership are quick and commonplace. Although these ownership judgments might appear simple on the surface, the principles which guide thinking about the ownership of data are less obvious. For example, embedded in the judgment that we own any piece of data shared with an app are intuitions about whether non-tangible data can be property at all; what types of data are and are not owned; and the nature of owners’ rights and responsibilities over their data. Looking at intuitions about data ownership can tell us about the psychology of ownership more generally, including how people come to see wholly new-in-time types of resources as potential forms of property, especially non-physical ones.

Thinking about ownership entails two key components (e.g., Nancekivell et al., 2019). First, it involves ownership attributions or deciding who owns what, and the second it involves considerations of ownership rights or attributions of whose in charge of what. One theory of ownership reasoning suggests that children’s thinking about ownership is coherent, theory-like, and structured (Nancekivell et al., 2019). This account predicts that children's thinking about the ownership of data should develop coherently. Namely, it predicts that at the same time point when children begin to attribute ownership over data should also be the same time point when they also attribute control or rights to the owner. In this way, the study of when children come to view data as property is informative for the coherence of ownership representations. Indeed, if children do not possess a coherent theory of ownership, they might show a more piecemeal developmental pattern wherein they might attribute ownership at an entirely different time point from control or vice versa.

One reason why it is an open question as to how children might reason about data ownership is because most of the work on children’s ownership understanding has focused on how children reason about tangible physical entities. However, there is relevant and important work that has examined children’s thinking about non-physical idea ownership and digital file ownership (Olson & Shaw, 2011; Shaw & Olson, 2015; Shaw et al., 2012). First, three studies have examined how children think about ideas (Olson & Shaw, 2011; Shaw & Olson, 2015; Shaw et al., 2012). This literature suggests that at around 6-years-old children can extend principles of physical ownership to non-physical ideas (Shaw et al., 2012). For example, like physical property, children view the first possessor of an idea as the owner of it. This literature also shows that unique factors like reputation govern thinking about idea ownership (Shaw & Olson, 2015). For example, people’s judgments of others’ actions are affected by whether the non-owner using an idea has attributed credit or not to the owner. Second, work on digital ownership shows that by 9-years children reason similarly to adults when reasoning about owners’ rights over digital files and more negatively evaluate file theft (Lee & Gelman, 2022). Together work on idea ownership and file ownership, suggests that children’s ability to reason about non-tangible property types likely emerges during middle childhood. It also suggests that there may be differences in how physical property and non-physical property are reasoned about.

Due to its focus on digital contexts, this study also builds on what is known about children’s thinking about digital rights which also changes during middle childhood. Five-year-olds know it is less acceptable to digitally track someone else’s location than one’s own location (Gelman et al., 2018). At around this age, children also view tracking of ingroup members as more acceptable than the tracking of outgroup members (Gelman et al., 2021). However, unlike adults, children view the tracking of others as generally acceptable (Gelman et al., 2018, 2021). Qualitative studies on digital safety and rights also find that with age ownership is more likely to arise as a theme in children’s conversations about their experiences online (Agesilaou & Kyza, 2022; Dowthwaite et al., 2020; Wang et al., 2023; Zhao et al., 2019). In sum, during middle childhood children become better at reasoning about the kinds of actions that violate people’s rights online, and qualitative studies suggest ownership reasoning could be related to this recognition.

As discussed, whether and when children view data as a kind of property is not known. However, prior work suggests that children reason in sophisticated ways about other related issues: who knows information, and whether it is acceptable to share different kinds of information like secrets. By 4-years-old children recognize that some types of information, namely generic facts, are more widely known than others (Cimpian & Scott, 2012; Diesendruck & Markson, 2011; Soley & Aldan, 2020). For example, preschoolers appreciate that others are more likely to know a generic fact like “hedgehogs eat hexapods” than a specific fact like “last night, this hedgehog ate a hexapod” (Cimpian & Scott, 2012). By 6-years-old, children understand that only some types of information, like information about a surprise birthday party, are likely to be ‘secrets’ (Anagnostaki et al., 2010). At around this same age, children think in sophisticated ways about the morality of information sharing (Kim et al., 2014; Liberman, 2020; Liberman & Shaw, 2018; Loke et al., 2011). For example, 4-year-olds think it is acceptable to share positive, but not negative information about someone else (Kim et al., 2014). Although related, this work does not examine the role of ownership in children’s thinking. For example, children might simply think sharing negative information is impermissible because it is interpersonally harmful.

## Present studies

In two studies, we examined the development of ownership attributions (who owns what), and rights over personal data (who should control the data). We asked children to judge who owns pieces of personal data a user willingly shared with an app (Study 1), and who should be in charge or control the data after it has been shared (Study 2). In both studies, we compare children’s thinking about two kinds of data: personal and general information. For example, children were told that Sally told the game “She lives in a house on Kirk Street.” (personal) and “Houses have kitchens.” (general). Notably, general information is not about Sally and so should not be viewed as owned (see prior work using general knowledge as a control; Shaw et al., 2012). Children in both studies had the option to select no-one as an option. Prior work has established that 4-year-old children understand ‘no-one’ as an ownership status (e.g., as in “It belongs to no-one.”; see Nancekivell et al., 2014). If children do not view information as a type of property, then they should select this answer.

An OSF project (https://osf.io/vt2hw/?view_only=1bd79ced2893478988ad3ea0ff9fa011) contains sample stimuli, analytic code, data analysis files, and additional figures.

## Study 1

In Study 1, we examined children’s ownership attributions (who owns what).

## Methods

### Participants

The final sample included 90 children ages 5-years-old to 10-years-old who were tested outdoors at children’s museums (41.3% female). The breakdown of the participants’ parent-reported racial/ethnic identities was as follows: 55% White, 28% Black, 8% Hispanic, 4% Asian, and 1% Native American. One additional 6-year-old was tested but excluded due to experimental error. The target sample size was 108 children, but testing stopped early due to COVID-19 limitations. Children were distributed across the target age range as follows: eighteen 5-year-olds, eighteen 6-year-olds, fifteen 7-year-olds, eighteen 8-year-olds, nine 9-year-olds, and twelve 10-year-olds. The study received research ethics committee (e.g. Institutional review board) approval.

### Materials and procedure

Children were told a story accompanied by pictures using physical flipbooks outdoors. The story was about a girl named Sally who was playing a game on her computer. Sally shares some pieces of information (data) with the game one by one. After each piece of information (see Table 1) is shared with the game, the experimenter told and then asked the child, *“The game has that information now. Who does it belong to: Sally, the game, or no-one?”.* If the child did not answer the question on the first try, the question was repeated once. Children were permitted to answer with any combination of responses (i.e., both game and Sally). An online game was used as it provided familiar context for children of different ages (see​ ​Hantrais et al., 2020).

**Table 1:** Information used in each study is presented by study and condition. The exact wording told to children is provided

Study	Personal Information	General Information
Study 1 & 2 *house item* *school item* *girl item*	Sally told the game she lives in a house on Kirk Street Sally told the game she goes to Smithson School Sally told the game she is a girl	Sally told the game houses have kitchens Sally told the game kids go to school Sally told the game some girls like rainbows
Study 2 only *name item*	Sally told the game she has a full name, Sally Anne Smith	Sally told the game some people have a first, middle, and last name

The study employed a within-subject block design where children were asked about three pieces of personal information and three pieces of general information. The order of the information within each block was presented in a fixed order (house, school, girl), but the presentation order of the information blocks (personal, general) was counterbalanced across versions. Information was designed to be as analogous as possible across conditions (see Table 1).

## Results

### Scoring and power

**Fig. 1:**
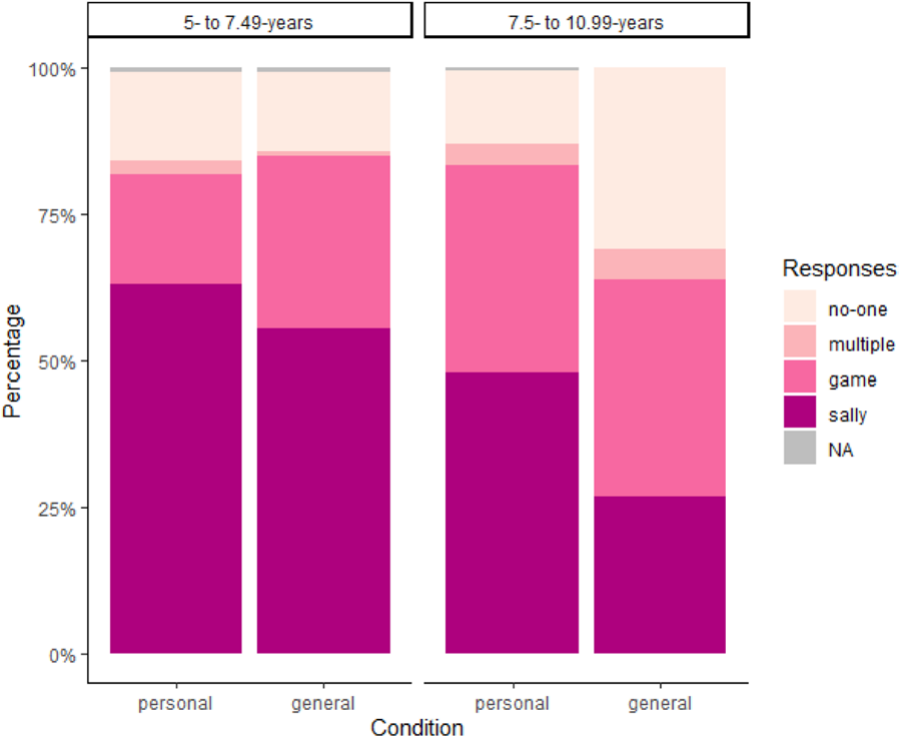
Distribution of all children’s responses by age group and condition

The main variable of interest was changes in Sally responses and so data were analyzed using a binomial regression where Sally = 1 and other responses = 0. The dataset includes three NAs which were also scored as 0 (there were no such responses in Experiment 2). Figure 1 displays all responses by condition. This figure suggests that including all response categories in our DV would have resulted in poor model fit as some non-Sally categories were infrequent in some conditions. Post hoc sample size analysis using pwr.f2.test suggests that a sample of 76 is adequate for a regression with three predictors (u = 3) and to detect a medium effect size (f2 = 0.15) with alpha of 0.05 and power of 0.8. Although binary regressions were conducted, linear models can provide a reasonable estimate for other kinds of generalized models (Cohen, 1988).

### Analytic plan

Regressions were built in R using lme4 package (Bates et al., 2015). We first ran an omnibus mixed binary regression which included a predictor of condition (personal, general), age (centered in months), and random effects modeled as (1 + condition|id). Effect sizes are reported as odds ratios (OR). Omnibus analyses treated age continuously; however, simple effects analyses and related visualizations unpacking age-effects treated age categorically. Specifically, we used a median split (median = 7.5-years) to create two groups: an older group (M = 9-years; *R* = 7.5 to 10.99 years; N = 46) and younger group (M = 6.2-years; *R* = 5.0 to 7.49-years; N = 44). For these simple effects analyses, separate mixed binary regressions were run for each age group with the predictor of condition (personal, general) and random effects modeled as (1 + condition|id). For all age calculations, if the month of birth was unknown, or the same month as the month tested, age in months was based on only the age in years (e.g., 6-years and 0 mos = 72 months). This occurred 14 times total (Study 1 and 2). For both studies, a graph showing data by item can be found in OSF.

### Analyses

**Fig. 2:**
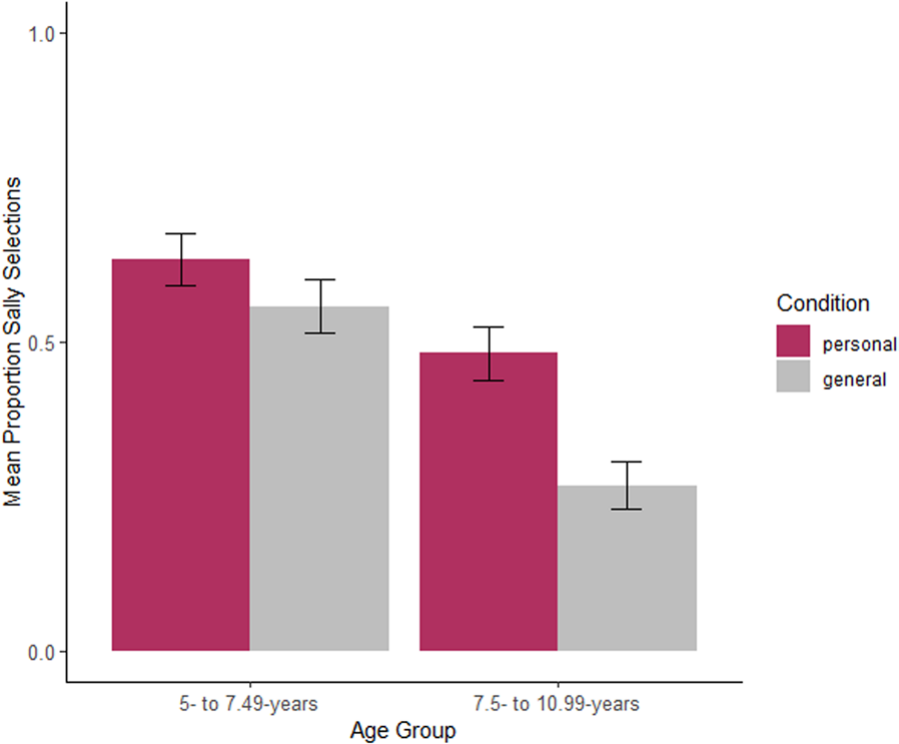
Distribution of children’s Study 1 Sally responses (as compared to all other responses) by condition (personal, general), and age group. Error bars represent standard error

Children selected Sally more often as the owner of the personal information than the general information, *b* =—0.52, *p* < .001, OR = 0.59. There was a main effect of age, *b* =—0.04, *p* < .001, OR = 0.96. These main effects were qualified by an age-by-condition interaction, *b* =—0.02, *p* = .033, OR = 0.98. As shown in Fig. 2, older children selected Sally more often as the owner of the personal information than the general information, *b* =—0.66, *p* = .012, OR = 0.52, but younger children selected Sally at similar rates across conditions, *b* =—0.41, *p* = .098, OR = 0.67.

## Study 2

Study 2 examined ownership rights attributions by asking children who (if anyone) should be “in charge of” the information.

## Methods

### Participants

In total, 128 children ages 5-years to 12-years were tested at various locations like science centers and farmers markets (50.7% female). The breakdown of the most frequent participants’ parent-reported racial/ethnic identities was as follows: 53% White, 13% First Nations/Métis, 5% Asian, and 5% multiple racial identities. 16% did not report their racial identity. Less than 3% of participants identified each of: Black, Muslim, Mennonite, and Ukrainian. Six additional children were tested but excluded for the following reasons: (3) parental interference, (1) language barriers, and (2) developmental disability.

### Materials and procedure

Children were told a story accompanied by pictures using a Qualtrics survey on a tablet. The study flow was similar to Study 1. In this study, during the introduction, children were told that it was their job to decide “*who should be in charge of the information.*” Although prior work has established that children understand the term “in charge” (e.g., Brey & Shutts, 2014; Dukler & Liberman, 2022), children were told as a precaution that: “*being in charge of information means picking who gets to use the information and who gets to tell it to other people. So, if you are in charge of information, you would get to decide who gets told it and who uses it.*” For this study, we used Qualtrics to randomize the order of information types as well as items within each block. To broaden test items, this study also included an additional non-location based item about names (see Table 1 for all items). At test, children had the same response options (e.g., Sally, the game, no-one).

## Results

The same analytic strategy was used as Study 1, but since this study had a larger age range, we created three age groups for the follow-up analyses: 5- to 7-years-olds (N = 48), 8- to 9-year-olds (N = 32), and 10- to 12-year-olds (N = 48).

### Analyses

**Fig. 3:**
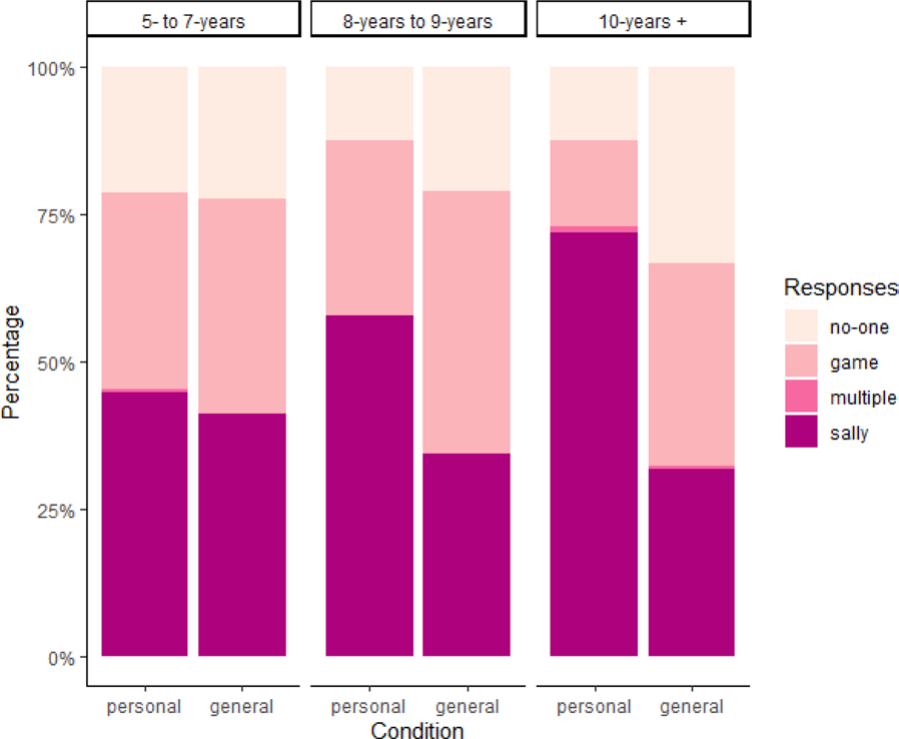
Distribution of children’s Study 2 responses by age group and condition (personal, general)

**Fig. 4:**
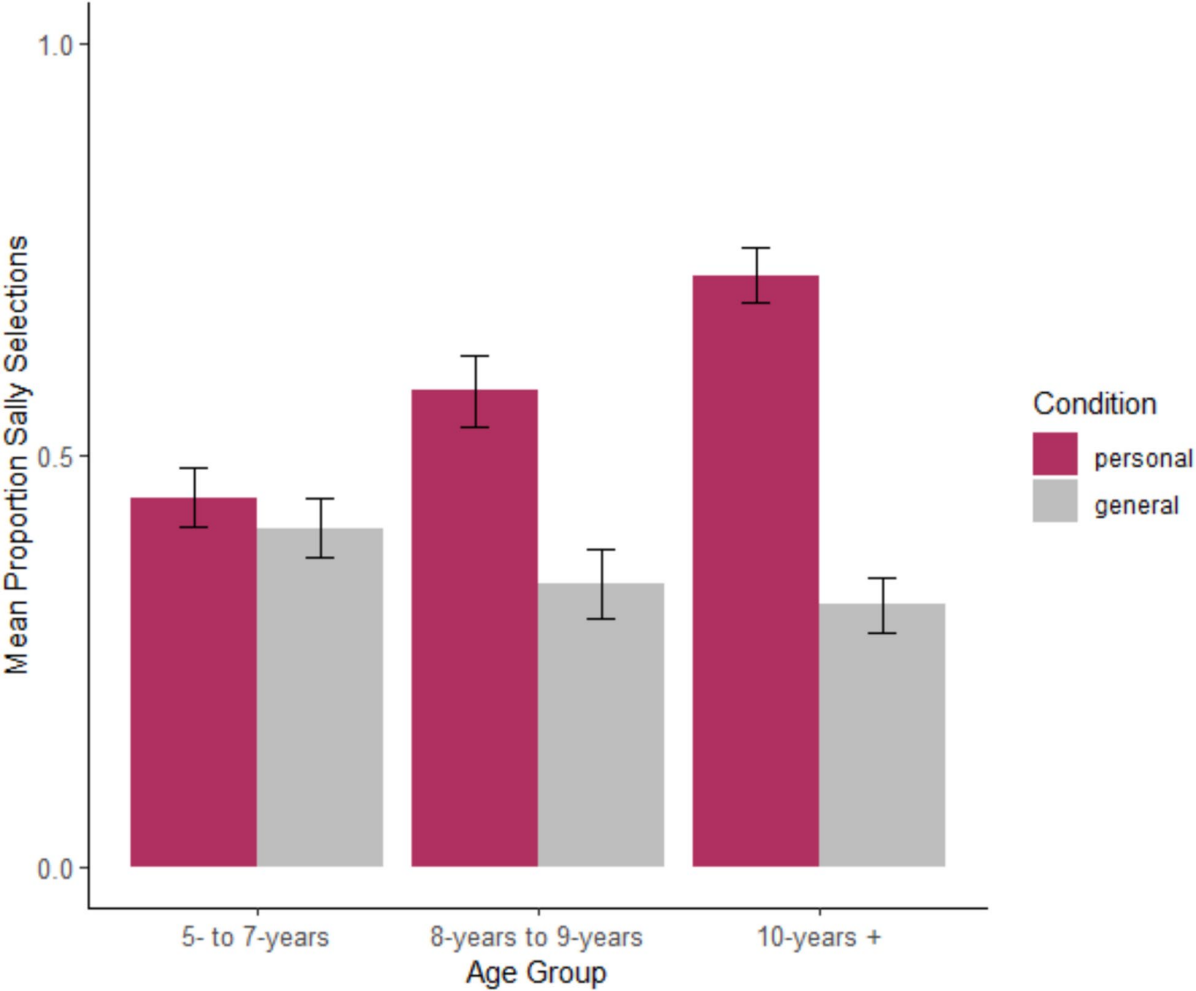
Distribution of children’s Study 2 Sally responses (as compared to all other responses) by condition (personal, general) and age group. Error bars represent standard error

As shown in Fig. 3, children selected Sally more often as ‘in charge’ of the personal information than the general information, *b* =—0.63, *p* < .001, OR = 0.53. Age did not affect the rate of Sally selections, *b* = 0.01, *p* = .12, OR = 1.01. As shown in Fig. 4, main effects were qualified by an age-by-condition interaction, *b* =—0.02, *p* < .001, OR = .98. The oldest children (10-years and older) selected Sally more often as the owner of the personal information than the general information, *b* = -1.25, *p* < .001, OR = 0.29, as did the middle age group (8- to 9-years), *b* = -0.68, *p* = .003, OR = .51. However, younger children (5- to 7-years) selected Sally at similar rates across conditions, *b* = -0.10, *p* = .41, OR = .91.

## General discussion

During middle childhood, children viewed users as more likely to be the owner of their personal data than the general information they share with apps and appreciated that users should be more “in charge” of or have rights over personal data than general information. Together, we found a similar developmental trajectory wherein at the same age children view data as more likely to be owned by the user they viewed her as more in charge of it.

Notably, across studies children were never told the differences between the two types of information. Instead, older children inferred this distinction for themselves. These findings suggest that as children age into middle childhood they come to appreciate that self-relevant information or personal data is likely to be a type of property with rights conferred therein. This supports extended self-accounts of ownership reasoning (e.g., Belk, 1988; Locke, 1978 [1690]).

Prior work suggests that young children possess a theory of ownership that should cross-cut property types and contexts (Nancekivell et al., 2019). As such, the present work contributes to what is known about ownership by mapping how children reason about a new abstract non-physical property type: personal data. The findings have bearing on the coherence of ownership representations in childhood. Namely, the same time point when children began to attribute ownership over data was also the same time point where they attributed control to the owner.

The findings in the present study build on prior work showing that middle childhood is a sensitive time period where children’s naive theories of physical ownership extend to other types of non-tangible property types. Specifically, prior shows that children extend their theory of ownership to intellectual property or ideas (Olson & Shaw, 2011; Shaw et al., 2012) and to digital files during middle childhood (Lee & Gelman, 2022). The similarities in developmental trajectory across studies suggest that experience might be necessary to trigger children to extend their theory of ownership to non-physical property types. In the context of digital property types, we suspect that with age children, as children’s social circles grow, they might be encountering more contexts online (and otherwise) where they have to make decisions about who gets to know their personal information. We suspect it is these experiences that lead them to realize that their personal information is a self-relevant entity for which they *get* to make decisions about. The same experience of making decisions about other kinds of digital assets like files, might also be necessary for children to reason effectively about other similar kinds of online non-physical property types (see Lee & Gelman, 2022). Supporting this idea, middle childhood is a timepoint at which children often use technology independently and time where they are making decisions about their data (e.g., Livingstone et al., 2019).

More work will be needed to fully understand the present findings as there is nuance in the data that we will next address. First, so far, we have discussed the pattern of young children’s judgments as suggesting that they did not view personal data as property. However, children judged that Sally owned her data at high rates in Study 1. Although our interpretation is the most conservative interpretation of their behavior, it could be that these high judgments do reflect some sort of deeper conceptual reasoning. A low-level interpretation of their behavior is that it reflects some sort of “Sally” response bias. But, one could instead interpret these Study 1 judgments as suggesting that children viewed Sally as *both* owning the personal data and the general facts she shared with the app. Namely, it could be that young children believe that users own any information they share with apps regardless of the content. This could be possible if children were using a heuristic like the first possessor heuristic to guide their judgments (e.g., Friedman & Neary, 2008). In our stories, Sally could be viewed as the first possessor of both kinds of information. To test this possibility, younger children’s judgments will need to be explored using an entirely different study frame that isolates first possession and that perhaps examines their beliefs in a non-transfer scenario.

Second, there was quite a bit of heterogeneity in children’s responses even at the older ages. For example, many older children viewed the game as owning the personal information. As mentioned, the present study relied on a transfer scenario to examine children’s judgments. This scenario was chosen because it is ecologically valid (i.e., we often reason about data ownership in the context of transfers), and because it was one of the few scenarios that would provide us with multiple reasonable competing response options. Namely, if the data have not been transferred, then access is automatically confounded with the ability to own it. Especially in the context of Study 2, how can one be in charge of an entity that it has no access to? Nonetheless, this choice in scenario likely introduced the opportunity for individual differences into the dataset. At a group level, a greater amount of children with age, viewed the personal data as more likely to be owned than general information, supporting the general developmental trends discussed. But, as mentioned children’s Sally judgments were far from ceiling. Future work should further investigate the heterogeneity in children’s judgments and whether they represent deeper conceptual differences in how children think about data ownership. For example, it could be that these individual differences represent heterogeneity in the degree to which children think that data can be transferred at all, with some viewing it as transferable and others not. This might be the case if personal data is viewed by some children as inalienable (similar to parts of bodies), but alienable by others (see work on alienability and ownership; Rose-Ackerman, 1985). What predicts individual differences in the view that data is inalienable, and its links to ownership reasoning will be an interesting and important area for future work.

## Conclusion

In sum, we found that around 8-years children view users as the owner of the personal data they share with apps, and appreciate that users should be “in charge” of or have rights over this personal data. This suggests that by 8-years-old children have the cognition in place to think about their data rights online. The developmental trajectory discovered in this report opens the door to explore many questions regarding the role of data ownership in children’s reasoning more broadly, including how children reason about the diverse moral and conventional contexts where data is shared.

## Data Availability

An OSF project (https://osf.io/vt2hw/?view_only=1bd79ced2893478988ad3ea0ff9fa011) contains sample stimuli, analytic code, data analysis files, and additional figures.
